# Prognostic role of CA‐125 in patients undergoing transcatheter aortic valve replacement: A systematic review and meta‐analysis

**DOI:** 10.1002/clc.24064

**Published:** 2023-07-17

**Authors:** Carlos Diaz‐Arocutipa, Jose Saucedo‐Chinchay, Mamas A. Mamas

**Affiliations:** ^1^ Vicerrectorado de Investigación Universidad San Ignacio de Loyola Lima Peru; ^2^ Department of Cardiology Hospital Nacional Edgardo Rebagliati Martins Lima Peru; ^3^ Keele Cardiovascular Research Group, Centre for Prognosis Research Keele University Keele UK

**Keywords:** carbohydrate antigen 125, systematic review, transcatheter aortic valve replacement

## Abstract

Transcatheter aortic valve replacement (TAVR) has become a widely used therapy for patients with severe aortic stenosis. Carbohydrate antigen 125 (CA‐125) is a promising biomarker in some cardiovascular diseases. This systematic review aims to assess the prognostic role of CA‐125 in patients undergoing TAVR. We searched electronic databases from inception to March 2023 to include cohort studies evaluating the association between preprocedural CA‐125 levels and mortality or heart failure (HF) readmission at 12 months in patients undergoing TAVR. We pooled crude (cHR) and adjusted hazard ratios (aHR) with their 95% confidence interval (CI) using a random‐effects model. The risk of bias was evaluated using the QUIPS tool. The certainty of the evidence was assessed using the GRADE approach. We included five cohort studies involving 1594 patients. Higher levels of CA‐125 were significantly associated with an increased risk of mortality or HF readmission using crude (cHR 2.79, 95% CI 1.45–5.36, *I* ^2^ =  72%) and adjusted (aHR 3.27, 95% CI 2.07–5.18, *I* ^2^ =  0%, high certainty) effect estimates compared with lower levels. Similarly, there was also associated with increased mortality using crude (cHR 2.68, 95% CI 1.99–3.60, *I* ^2^ =  0%) and adjusted (aHR 2.17, 95% CI 1.54–3.07, *I* ^2^ =  0%, high certainty) effect estimates. The risk of bias varied between low to moderate across studies. Our meta‐analysis suggests that CA‐125 has incremental prognostic value in patients undergoing TAVR. Further studies are needed to determine the clinical utility of CA‐125 in guiding treatment decisions in this population.

## Graphical abstract

Prognostic role of CA‐125 in patients undergoing TAVR. CA‐125, carbohydrate antigen 125; CI, confidence interval; TAVR, transcatheter aortic valve replacement.

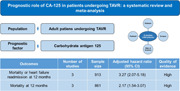

## 1 INTRODUCTION

Transcatheter aortic valve replacement (TAVR) is an alternative to surgical aortic valve replacement for patients with severe aortic stenosis who are at moderate or high surgical risk or selected low‐risk patient groups.^1^ Although TAVR has been shown to have favorable outcomes in these patients, there is still a need for accurate prognostic markers to help identify those who may benefit most from this intervention.^1^ One such biomarker that has garnered interest in recent years is carbohydrate antigen 125 (CA‐125).^2^

CA‐125 is a glycoprotein antigen that is primarily used as a tumor marker for ovarian cancer.^3^ However, it has also been found to be elevated in a range of other malignancies as well as in nonneoplastic conditions such as cardiovascular disease.^4^ Recent studies have assessed the utility of CA‐125 as a prognostic marker in patients undergoing TAVR, although data have been conflicting.^5^, ^6^ Therefore, this systematic review and meta‐analysis aim to synthesize the available evidence on the prognostic role of CA‐125 in patients undergoing TAVR.

## 2 MATERIALS AND METHODS

This systematic review was reported according to the 2020 Preferred Reporting Items for Systematic Reviews and Meta‐Analysis Protocols (PRISMA) statement.^7^

### 2.1 Search strategy

We searched the following electronic databases: PubMed, Embase, Scopus, and Web of Science. The searches covered the period from inception until March 2023. We did not limit the search based on publication date or language. We used the following search terms with their combinations, but not limited to CA‐125 and TAVR. We also conducted a citation search of all included studies to identify other potentially eligible studies. The detailed search strategy for all databases can be found in Supporting Information: Table S1.

### 2.2 Eligibility criteria

The inclusion criteria were as follows: (i) prospective or retrospective cohort studies that included adult patients undergoing TAVR, (ii) studies that measured CA‐125 in blood at least 24 hours before the TAVR procedure, and (iii) studies that reported data on crude and adjusted effect estimates for at least one assessed outcome at any follow‐up times. Case reports, case series, cross‐sectional studies, case–control studies, editorials, conference abstracts, and reviews were excluded.

### 2.3 Study selection

We downloaded all articles from electronic search to EndNote™ 20 and removed duplicate records. We uploaded all unique articles to Rayyan (https://rayyan.qcri.org/) for the study selection process. Titles and abstracts were independently screened by two review authors to identify relevant studies. Likewise, the same two review authors independently examined the full‐text of selected studies and registered reasons for the exclusion. Any disagreement was resolved by consensus.

### 2.4 Outcomes

The primary outcome was mortality or heart failure readmission at 12 months and the secondary outcome was mortality at 12 months. We used study definitions for all outcomes.

### 2.5 Data extraction

For each eligible study, two review authors independently extracted the data using a standard data extraction form based on the CHARMS‐PF checklist.^8^ Disagreements were resolved by consensus. We extracted the following information: study characteristics (year of publication, country, study design, study period, and study site), population characteristics (inclusion criteria, sample size, age, sex, comorbidities, TAVR characteristics, pre‐TAVR left ventricular ejection fraction [LVEF], pre‐TAVR aortic valvular area, pre‐TAVR mean gradient, and length of hospital stay), description of prognostic factor (timing of CA‐125, method of CA‐125 assessment, and cut‐off levels of CA‐125), and outcomes (definition, effect estimates, and follow‐up duration).

### 2.6 Risk of bias assessment

We used the Quality in Prognosis Studies (QUIPS) tool for prognostic factor studies to assess the risk of bias in included studies.^9^ This tool includes the following six domains: study participation, study attrition, prognostic factor measurement, outcome measurement, adjustment for other prognostic factors, and statistical analysis and reporting. Each study was judged as having high, moderate, and low risk of bias. Two review authors independently performed assessments and any disagreements were resolved by consensus.

### 2.7 Assessment of certainty of the evidence

To assess the certainty of the evidence across all studies related to a prognostic factor, we used the Grading of Recommendations, Assessment, Development, and Evaluation (GRADE) approach for adjusted effect estimates.^10^ These judgments take into consideration the following domains: risk of bias, indirectness, inconsistency, imprecision, publication bias, large effect, dose–response, and plausible confounding. We generate the summary of findings table and rated the certainty of the evidence as high, moderate, low, or very low for each outcome. Absolute risks with and without predictors were calculated from the adjusted effect sizes using Foroutan's method.^10^ We chose a 5% risk difference as our clinical importance threshold to determine if there is a prognostic value of CA‐125 across all outcomes.

### 2.8 Statistical analysis

We used a random‐effects model for all meta‐analyses. The between‐study variance (τ^2^) was estimated using the Paule–Mandel estimator. We pooled crude (cHR) and adjusted (aHR) hazard ratios with their 95% confidence interval (CI) for all outcomes. In addition, we also pooled the crude risk ratio (cRR) using the data from the published studies to construct 2 × 2 tables. Statistical heterogeneity was assessed through visual inspection of forest plots (looking for the consistency of point estimates and the extent of overlap in CIs) and *I*² statistic (threshold > 60%). Publication bias was evaluated only if 10 or more studies were available. All meta‐analyses were performed using the *meta* package from R 4.2.3 (R Core Team). A two‐tailed *p* < .05 was considered statistically significant.

## 3 RESULTS

### 3.1 Study selection

**Figure 1:**
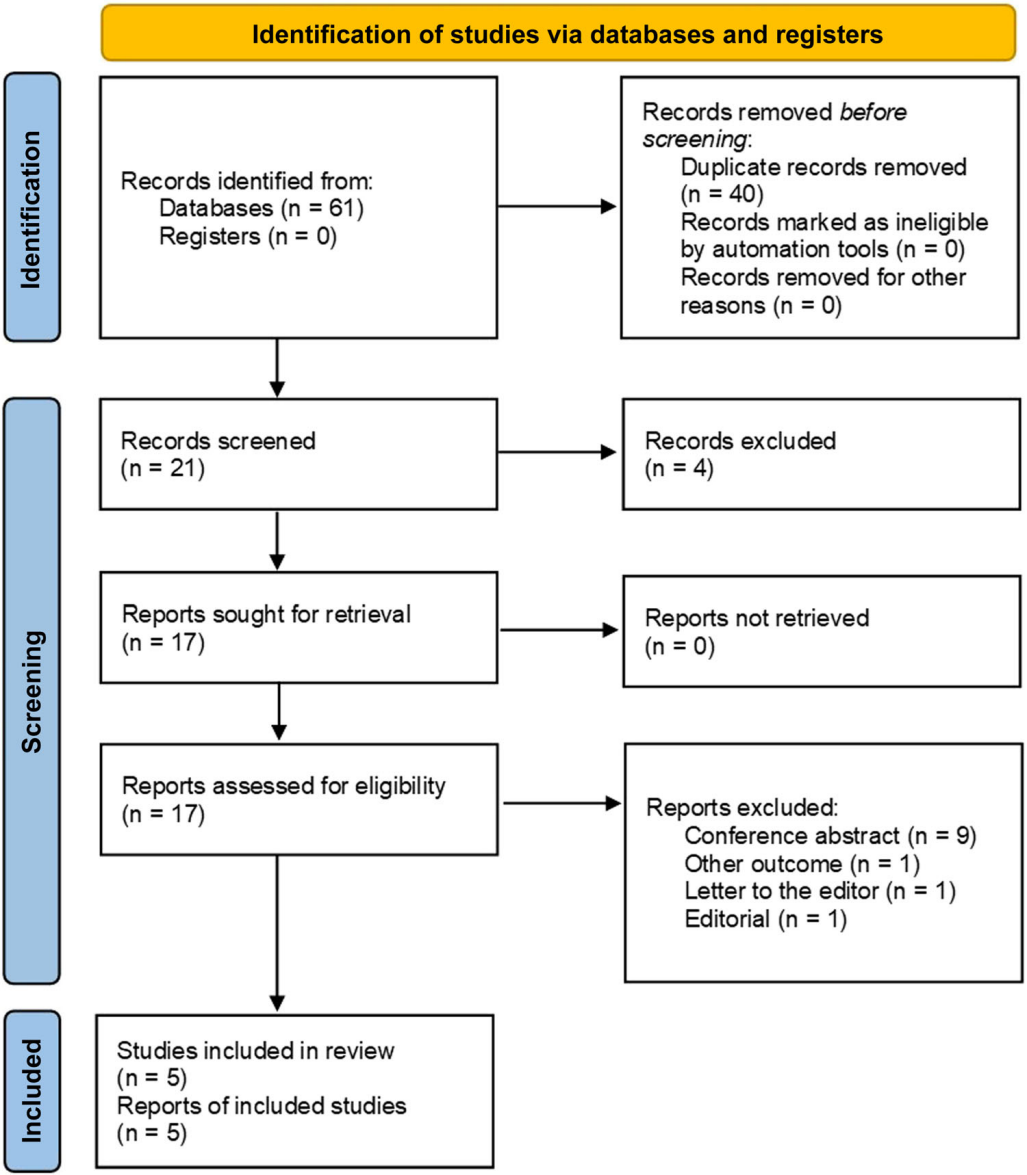
Flow diagram of study selection.

The electronic search yielded 61 studies. After screening titles and abstracts, we obtained 17 study reports for full‐text review. After the full‐text screening, 12 studies were excluded for the following reasons: conference abstract (*n* = 9), other outcomes (*n* = 1), letter to the editor (*n* = 1), and editorial (*n* = 1). Finally, five cohorts detailing associations between CA‐125 and outcomes in patients undergoing TAVR were selected (Figure 1).^5^, ^6^, ^11^, ^12^, ^13^

### 3.2 Study characteristics

The main characteristics of the studies were summarized in Table 1. A total of 1366 patients were included (sample sizes ranged from 31 to 439). The mean age ranged from 76.8 to 85 years and 48% of patients were male. Most studies were conducted in Germany (75%). The median length of hospital stay ranged between 3 and 8 days across studies. The most common comorbidities were hypertension (83%), hyperlipidemia (67%), and coronary artery disease (64%). The type of TAVR implanted was reported in only two studies,^11^, ^12^ the most commonly used being the Edwards SAPIEN XT prosthesis (80%). The most frequent TAVR implantation route was transfemoral (91%). The indication for TAVR was severe aortic stenosis in all studies. The cut‐off points of CA‐125 to define high or low levels varied between 18.2 and 35 U/mL across studies. The association between CA‐125 levels and some patient characteristics was reported in only two studies.^11^, ^14^ Ayhan et al.^11^ found a significant association only with LVEF, with higher CA‐125 levels (>35 U/mL) in patients with reduced LVEF (mean 61% vs. 37.6%, *p* < .001). Husser et al.^14^ reported significant association between elevated CA‐125 levels (>15.7 U/mL) with male sex (61% vs. 47%, *p* = .039), higher logistic EuroSCORE score (median 18 vs. 11, *p* < .001), renal failure (36% vs. 21%, *p* = .016), NYHA functional class III/IV (97% vs. 69%, *p* < .001), and LVEF < 50% (32% vs. 9%, *p* < .001).

**Table 1:** Characteristics of included studies.

Reference	Sample size	Population	Cut‐off levels for CA‐125	Follow‐up duration	Length of hospital stay, days	Age, years	Male	Comorbidities	LVEF, %	AVA, cm^2^
Ayhan^11^	31	Patients with severe symptomatic aortic stenosis (high risk or inoperable)	35 U/mL	30 days and 9 months	6.8 ± 4.8^a^	76.8 ± 6^a^	42%	Hypertension (71%), diabetes (32%), CAD (90%), PAD (39%), AF (29%), hyperlipidemia (48%)	50.9 ± 17.3%^a^	0.6 ± 0.1^a^
Husser^12^	422	Consecutive patients who underwent TAVR for severe aortic stenosis	30 U/mL	59 (34–107)^b^ weeks	8 (7–13)^b^	79 ± 6^a^	47%	Hypertension (72%), diabetes (32%), COPD (14%), CAD (39%), PAD (14%), AF (34%)	≤35% (7%), >35% (93%)	0.71 ± 0.2^a^
Rheude^5^	363	Patients with severe aortic stenosis who underwent transfemoral TAVR	18.4 U/mL	12 months	5 (4–6)^b^	81 ± 6^a^	54%	Hypertension (91%), diabetes (29%), COPD (14%), CAD (69%), PAD (13%), AF (26%), hyperlipidemia (73%)	≤35% (11%), >35% (89%)	0.73 ± 0.21^a^
Rheude^13^	439	Consecutive patients with symptomatic severe aortic stenosis undergoing transfemoral TAVR	18.4 U/mL	371 (219–402)^b^ days	5 (4–6)^b^	81 (77–85)^b^	55%	Hypertension (91%), diabetes (26%), COPD (16%), CAD (74%), PAD (13%), AF (41%), hyperlipidemia (79%)	≤35% (9%), >35% (91%)	Not reported
Romeo^6^	111	Patients with severe aortic stenosis undergoing transfemoral TAVR	18.2 U/mL	12 months	3 (3–4)^b^	85 (80–88)^b^	43%	Hypertension (90%), diabetes (16%), COPD (13%), CAD (46%), PAD (32%), AF (28%), hyperlipidemia (68%)	60% (55–64)^b^	0.7 (0.6–0.9)^b^

Abbreviations: AF, atrial fibrillation; AVA, aortic valve area; CAD, coronary artery disease; COPD, chronic obstructive pulmonary disease; LVEF, left ventricular ejection fraction; PAD, peripheral artery disease; TAVR, transcatheter aortic valve replacement.

^a^ Mean ± standard deviation.

^b^ Median (interquartile range).

### 3.3 Risk of bias assessment

**Figure 2:**
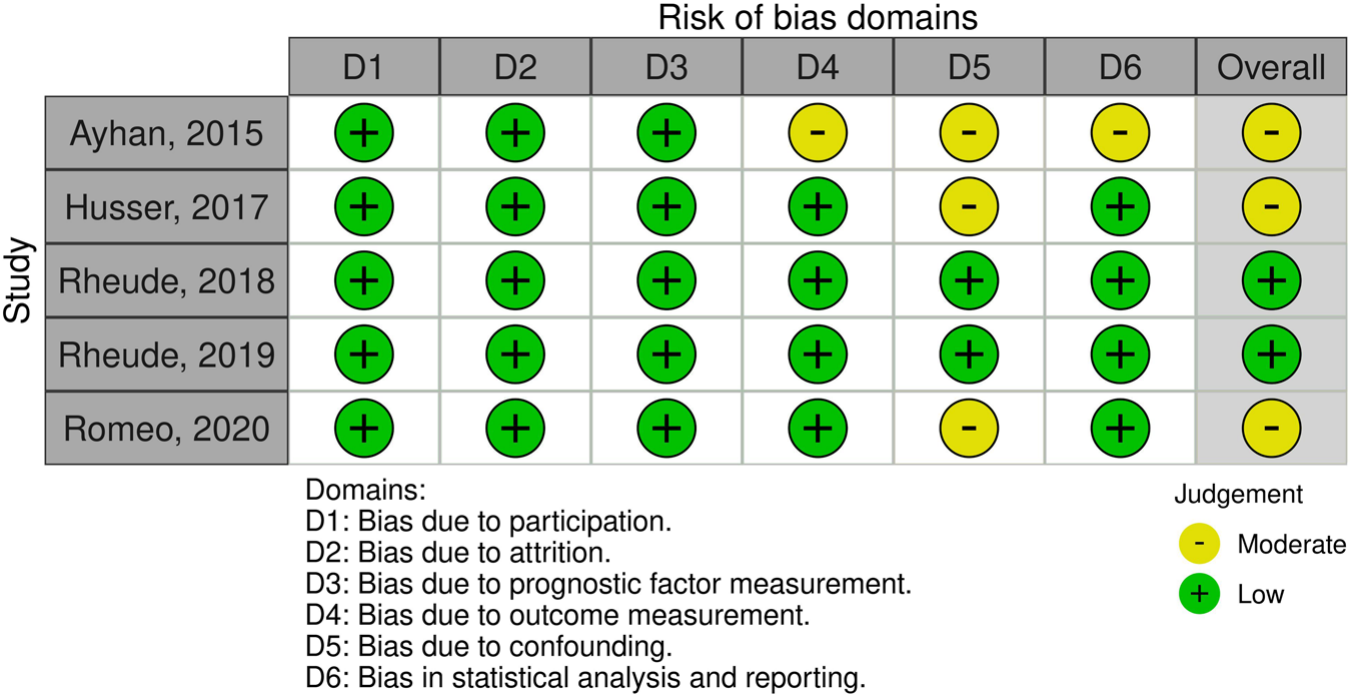
Risk of bias assessment according to the Quality in Prognosis Studies (QUIPS) tool.

We provide a summary of the risk of bias results for each of the domains according to the QUIPS tool in Figure 2. Two studies were rated at low risk of bias and three studies at moderate risk of bias. The most frequent source of bias related to adjustment for other prognostic factors.

### 3.4 Mortality or HF readmission

**Figure 3:**
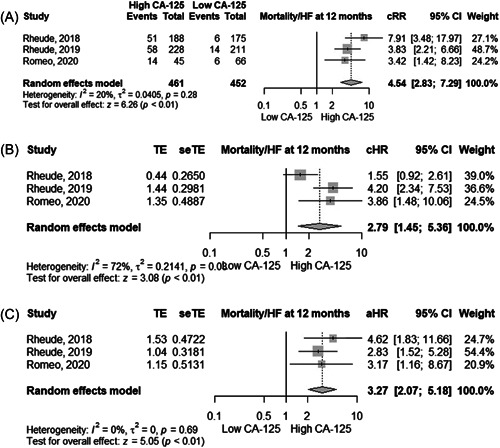
Forest plot showing the (A) crude relative risk, (B) crude hazard ratio, and (C) adjusted hazard ratio between CA‐125 levels and mortality or heart failure readmission at 12 months in patients undergoing TAVR. aHR, adjusted hazard ratio; cHR, crude hazard ratio; cRR, crude risk ratio; CI, confidence interval; TAVR, transcatheter aortic valve replacement.

In three studies, the absolute risk of mortality in patients with high and low levels of CA‐125 was 23.8% and 8%, respectively. High levels of CA‐125 were significantly associated with a higher risk of mortality or HF readmission at 12 months using cRR (4.54, 95% CI 2.83–7.29, *I* ^2^ =  20%), cHR (2.79, 95% CI 1.45–5.36, *I* ^2^ =  72%), and aHR (3.27, 95% CI 2.07*‐*5.18, *I* ^2^ =  0%, high certainty) (Figure 3 and Table 2).

**Table 2:** Summary of findings table.

Prognostic factor	Study results and measurements	Absolute effect estimates		Certainty in effect estimates	Plain text summary
		Baseline	With predictor		
*Mortality or HF readmission at 12 months*					
CA‐125 (High vs. Low)	Adjusted HR 3.27 (95% CI 2.07–5.18) Based on data from 913 patients in three studies	80 per 1000	238 per 1000	High	High CA‐125 levels increase 12‐month mortality or HF readmission
		Difference: 158 more per 1000 (95% CI 92 more–222 more)			
*Mortality at 12 months*					
CA‐125 (High vs. Low)	Adjusted HR 2.17 (95% CI 1.54–3.07) Based on data from 861 patients in two studies	78 per 1000	161 per 1000	High	High CA‐125 levels increase 12‐month mortality
		Difference: 83 more per 1000 (95% CI 41 more–131 more)			

Abbreviations: CA‐125, carbohydrate antigen 125; CI, confidence interval; HF, heart failure; HR, hazard ratio.

### 3.5 Mortality

**Figure 4:**
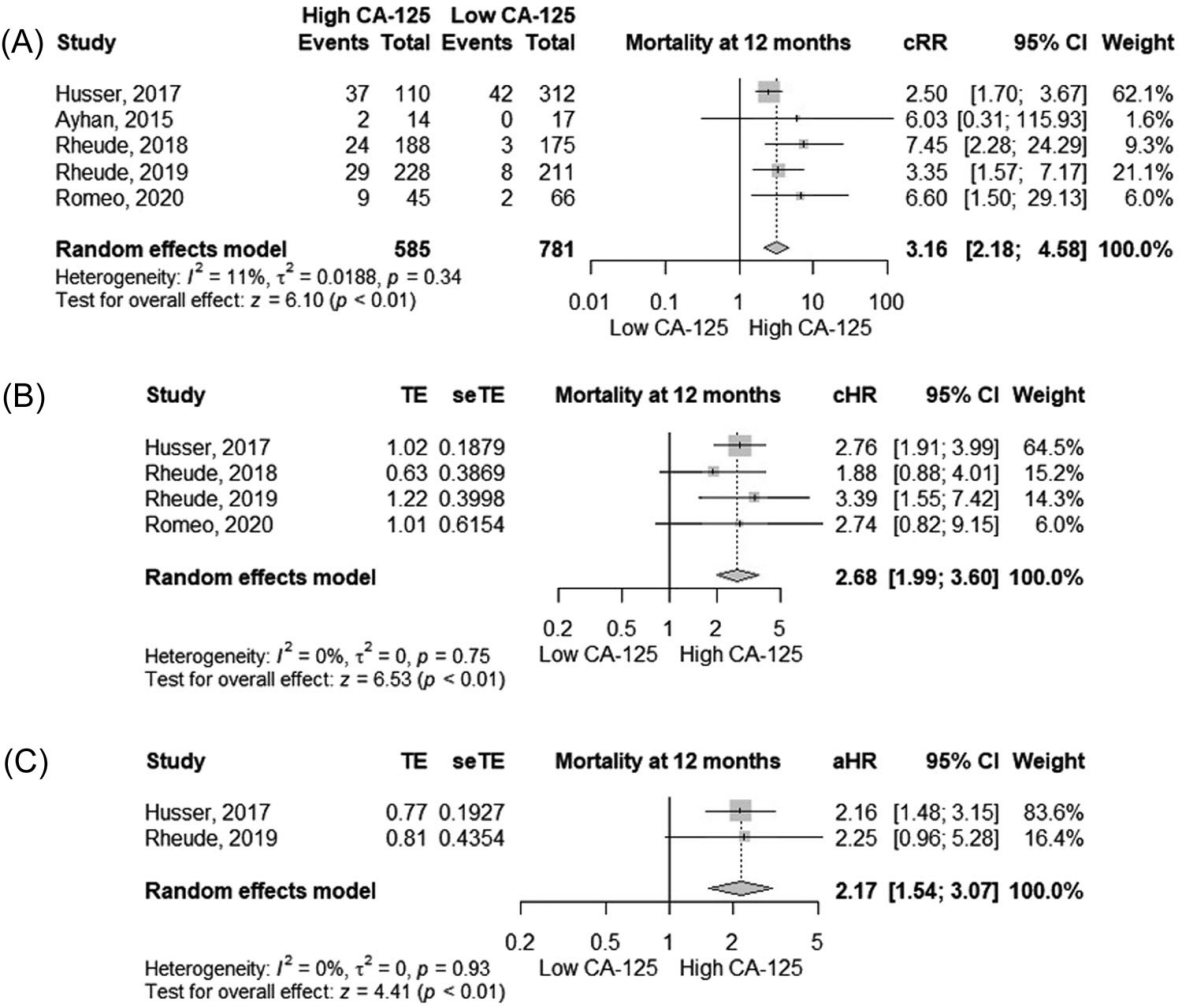
Forest plot showing the (A) crude relative risk, (B) crude hazard ratio, and (C) adjusted hazard ratio between CA‐125 levels and mortality at 12 months in patients undergoing TAVR. aHR, adjusted hazard ratio; cHR, crude hazard ratio; CI, confidence interval; cRR, crude risk ratio; TAVR, transcatheter aortic valve replacement.

In five studies, the absolute risk of mortality in patients with high and low levels of CA‐125 was 16.1% and 7.8%, respectively. High levels of CA‐125 were significantly associated with a higher risk of mortality at 12 months using cRR (3.16, 95% CI 2.18–4.58, *I* ^2^ =  11%), cHR (2.68, 95% CI 1.99–3.60, *I* ^2^ =  0%), and aHR (2.17, 95% CI 1.54–3.07, *I* ^2^ =  0%, high certainty) (Figure 4 and Table 2).

## 4 DISCUSSION

This systematic review shows, with a high degree of certainty, that high preprocedural levels of CA‐125 were significantly associated with poor survival or higher HF readmission at 12 months in patients undergoing TAVR using crude and adjusted effect estimates. The estimation of absolute risks demonstrates that patients with high CA‐125 levels showed an absolute risk increase of more than 5% (15.8% for mortality or HF readmission and 8.3% for mortality) in comparison with low CA‐125 levels. The risk of bias was low or moderate across the studies.

The pathophysiological basis for the relationship between high CA‐125 levels and clinical outcomes in patients undergoing TAVR is not fully understood. However, some possible mechanisms could explain this association. CA‐125 is a well‐established biomarker for ovarian cancer, and its elevation has been linked to various pathological processes, including inflammation, tissue injury, and fibrosis.^3^ In the context of TAVR, it has been suggested that elevated CA‐125 levels may reflect increased inflammation and myocardial injury, which are associated with adverse outcomes in patients with severe aortic stenosis.^13^ Moreover, CA‐125 is involved in the regulation of cell adhesion and angiogenesis,^15^, ^16^ processes that are crucial for tissue repair and remodeling after TAVR. Additionally, CA‐125 may also be a marker of cardiac dysfunction, as it has been shown to correlate with left ventricular mass and ejection fraction in patients with heart failure.^17^ Hence, the presence of high CA‐125 levels in patients undergoing TAVR may indicate a more severe disease state and increased risk of adverse outcomes.

To the best of our knowledge, this is the first systematic review that evaluates the prognostic role of CA‐125 levels in patients undergoing TAVR. Previous studies have investigated the role of biomarkers in predicting outcomes after TAVR. For instance, the systematic reviews by Takagi et al.,^18^ Li et al.,^19^ and Takagi et al.^20^ identified several biomarkers that were associated with increased mortality after TAVR, including natriuretic peptides, troponins, and high‐sensitivity C‐reactive protein, respectively. Several prognostic models have been proposed to predict outcomes in patients undergoing TAVR, including the Society of Thoracic Surgeons (STS) score, EuroSCORE, and EuroSCORE II.^21^ However, these models have limitations in predicting individual patient outcomes, especially in high‐risk patients. Our study adds to this body of evidence by identifying CA‐125 as a potential prognostic biomarker for risk stratification in patients undergoing TAVR. CA‐125 may provide additional information and could be used in conjunction with existing models to improve risk prediction. However, further research is needed to determine the optimal cut‐off points for CA‐125 levels and to validate its use in clinical practice. In addition, it is also necessary to investigate the potential benefits of targeting CA‐125 levels to improve patient outcomes after TAVR.

Our review has some limitations that should be acknowledged. First, only five studies were included in the analysis, which may have limited the generalizability of the findings. Second, the heterogeneity in cut‐off points used to define high or low CA‐125 levels across studies may have contributed to variability in effect estimates. Finally, while the included studies adjusted for several prognostic factors, the set of adjustment factors varied between studies (Supporting Information: Table S2).

## 5 CONCLUSIONS

In conclusion, our meta‐analysis suggests that CA‐125 is a useful prognostic marker in patients undergoing TAVR, with high levels being associated with an increased independent risk of mortality or HF readmission at 12 months. Clinicians may consider incorporating CA‐125 levels into their risk stratification algorithms to optimize patient management and outcomes.

## AUTHOR CONTRIBUTIONS

*Concept/design*: Carlos Diaz‐Arocutipa and Jose Saucedo‐Chinchay. *Data acquisition*: Carlos Diaz‐Arocutipa and Jose Saucedo‐Chinchay. *Data analysis/interpretation*: Carlos Diaz‐Arocutipa, Jose Saucedo‐Chinchay, and Mamas A. Mamas. *Drafted the article*: Carlos Diaz‐Arocutipa. *Critically revised the article*: Jose Saucedo‐Chinchay and Mamas A. Mamas. *Approved the article*: Carlos Diaz‐Arocutipa, Jose Saucedo‐Chinchay, and Mamas A. Mamas.

## CONFLICT OF INTEREST STATEMENT

The authors declare no conflict of interest.

## Supporting information

Supporting information.

## Data Availability

The data that support the findings of this study are available from the corresponding author upon reasonable request.
