# Supplementary material for: Prognostic role of CA‐125 in patients undergoing transcatheter aortic valve replacement: A systematic review and meta‐analysis
Source: Clin Cardiol. 2023 Jul 17;46(10):1129–36. doi: 10.1002/clc.24064 (PMC10577531; doi:10.1002/clc.24064)
Supplement: Supplementary file 1 — Supporting information. [file CLC-46-1129-s001.docx]

**SUPPLEMENTARY DATA**

**Table S1. Search strategy**

| **PubMed**  ("CA-125 Antigen"[mesh] OR "ca 125"[tiab] OR ca125[tiab] OR "antigen 125"[tiab]) AND ("transcatheter aortic valve replacement"[mesh] OR "transcatheter aortic valve"[tiab] OR TAVR[tiab] OR TAVI[tiab]) |
| --- |
| **Embase**  ('ca 125 antigen'/exp OR 'ca 125':ti,ab,kw OR 'ca125':ti,ab,kw OR 'antigen 125':ti,ab,kw) AND ('transcatheter aortic valve implantation'/exp OR 'transcatheter aortic valve':ti,ab,kw OR 'tavr':ti,ab,kw OR 'tavi':ti,ab,kw) |
| **Scopus**  TITLE-ABS-KEY ( ( "ca 125" OR ca125 OR "antigen 125" ) AND ( "transcatheter aortic valve" OR tavr OR tavi ) ) |
| **Web of Science**  TS=( ( "ca 125" OR ca125 OR "antigen 125" ) AND ( "transcatheter aortic valve" OR tavr OR tavi ) ) |

**Table S2. Adjusted variables in the multivariable regression model for hazard ratio estimation**

| **Study** | **Adjusted variables** |
| --- | --- |
| **Mortality or HF readmission** | |
| Rheude, 2018 | Age, logistic EuroSCORE, NYHA III/IV, MI, PAD, anemia, AF, creatinine, CRP, LVEF<=35%, PAP>60, mean transaortic gradient, NT-proBNP |
| Rheude, 2019 | Age, logistic EuroScore, previus myocardial infarction, previous cancer, AF, mitral regurgitation III/IV, pulmonary hypertension, hemoglobin, creatinine clearance, NT-proBNP, mean transvalvular gradient, LVEF<35%, CRP |
| Romeo, 2020 | STS score, frailty, PAP≥60 mmHg, elevated CRP |
| **Mortality** | |
| Husser, 2013 | Logistic EuroSCORE, NYHA III/IV, device success |
| Husser, 2017 | EuroScore, diabetes, AF, previous pacemaker, elevated NYHA |
| Rheude, 2019 | Age, logistic EuroSCORE, previus myocardial infarction, previous cancer, AF, mitral regurgitation III/IV, pulmonary hypertension, hemoglobin, creatinine clearance, NT-proBNP, mean transvalvular gradient, LVEF<35%, CRP |

Abbreviations: HF, heart failure; NYHA, New York Heart Association; PAD, perfipheral artery disease; AF, atrial fibrillation; CRP, C-reactive protein; LVEF, left ventricular ejection fraction; PAP, pulmonary artery pressure; STS, Society of Thoracic Surgeons.
